# Staff’s insights into fall prevention solutions in long-term care facilities: a cross-sectional study

**DOI:** 10.1186/s12877-023-04435-7

**Published:** 2023-11-13

**Authors:** Neah Albasha, Catriona Curtin, Ruth McCullagh, Nicola Cornally, Suzanne Timmons

**Affiliations:** 1 Center for Gerontology and Rehabilitation, School of Medicine, University College Cork, Cork, Ireland; 2 Rehabilitation Department, College of Health and Rehabilitation Sciences, Princess Nourah Bint Abdulrahman University, Riyadh, Saudi Arabia; 3 Discipline of Physiotherapy, School of Clinical Therapies, University College Cork, Cork, Ireland; 4 School of Nursing and Midwifery, University College Cork, Cork, Ireland

**Keywords:** Fall prevention, Older person, Long-term care, Staff, Current practices, Solutions, Education

## Abstract

**Background:**

Falls are one of the most common and serious health issues in long-term care facilities (LTCFs), impacting not just residents, but staff and the healthcare system. This study aimed to explore LTCF staff’s current practices around falls prevention, and their suggested solutions for better falls prevention.

**Methods:**

In the southwest of Ireland, a descriptive cross-sectional study was conducted in 13 LTCF sites, across a range of provider types and facility sizes. A survey, measuring staff knowledge, skills and attitudes, was distributed in physical and online formats. Staff suggestions for prioritising fall and fall-related injury prevention activities, and current staff practices regarding fall incidents were also sought. Content analysis was used to analyse responses, mapping categories and subcategories to the refined theoretical domains framework (TDF) and to an existing fall prevention guideline.

**Results:**

There were 155 respondents (15% response rate), from staff of the LTCFs. Environmental reviews and modifications (aligned to the TDF environmental context and resource domain) were the most common suggestions for preventing both falls and fall-related injuries. Other common suggestions for preventing falls were staff education, monitoring of residents, and using alarm/calling systems, while few staff members, across all roles, reported assessing residents, exercises, reviewing medications, and vitamin D supplements. For preventing fall-related injuries, suggestions included protective equipment, hip protectors and alarm/calling systems. Staff used a standardised approach when responding to a fall incident, with intensive and holistic post-fall control measures. HCAs focussed on transferring residents safely, while nurses of all grades focused more on post-fall assessment. Respondents believed that staff education, communication, increasing staffing levels and enhancing specialist care could support their practice.

**Conclusion:**

Noting the low response rate, the results suggest an awareness gap regarding some evidence-based, resident-focussed falls prevention solutions, such as pro-active fall-risk assessment, exercise, medication review, and Vitamin D supplements. These aspects should be included in future fall prevention education programmes in LTCFs.

**Supplementary Information:**

The online version contains supplementary material available at 10.1186/s12877-023-04435-7.

## Background

Falls are very common in older people, contributing significantly to mortality and morbidity [1]. Falls impact 684,000 people annually, making it the second leading cause of death globally [2]. Residents in long-term care facilities (LTCF) are more likely to fall and sustain fall-related injuries, since they are frail and vulnerable, with co-morbidities, disabilities and decreased functional capacity [3–7]. In LTCFs, up to 50% of older residents experience falls yearly, and 40% fall recurrently [8, 9]. Their incidence of falls is three times higher than their peers in community settings, at 1.7 falls per person per year [3, 4]. Thus, all residents in LTCFs are at risks of falls, such that stratifying their ‘risk of falls’ is not relevant, and instead the focus is on falls prevention for all [10].

Additionally, 10 to 25% of falls in LTCF result in significant injuries, compared to 5% in the community [3–5]. Based on the Public Health Agency of Canada, falls in LTCFs cause 6,000 to 12,884 cases of hospital admissions annually for older people aged 65 or older, with a typical stay of 12 to 20 days [11]. According to the National Institute for Clinical Excellence (NICE), the annual cost of falls and fall-related injuries in the UK for older people aged 65 or older who are at high risk of falling is estimated to be 2.3 and 1.7 billion pounds sterling, respectively [1].

Apart from the economic cost, falls cause physical injury, especially hip fracture, estimated at 3–5% of cases yearly among resident aged 65 and above [12, 13], and hence pain and reduced function, but also fear of falling, depression and loss of confidence and independence [8, 9]. The nature of falls is complex, because they involve multifaceted risk factors, both intrinsic physical (e.g., ageing, chronic disease, cognitive impairments) and psychological issues (e.g., fear of falling), and extrinsic, i.e., environmental hazards [14–16]. These risk factors are strongly predictive of falls and potentially controllable [3].

Many fall prevention interventions exist, whether single or multifactorial [4, 17–19]. Multifactorial interventions have been proven to be effective at reducing falls in LTCFs, whereas single intervention effects are inconsistent [4, 17, 18]. Additionally, many guidelines exist for multifactorial falls risk assessment (i.e., identifying any factors that increase the risk of falls for the individual older person) and preventing falls and fall-related injuries [1, 6, 20, 21]. Staff should be able to conduct multifactorial falls risk assessments and identify effective fall prevention interventions [22], for implementation by the multidisciplinary team (including nurses, healthcare assistants, physiotherapists, etc.) [23, 24]. Identifying a resident’s risk factors for falling allows staff to intervene and prevent fall episodes [25], noting the challenges that exist in the physical environment, resident-specific characteristics, and ingrained care practice [26].

Falls prevention is a key element of patient safety and a critical clinical quality indicator in healthcare institutions [27]. Their knowledge and skills significantly contribute to adopting a comprehensive approach to fall evaluation and prevention. However, LTCF staff are known to be concerned about their capacity to handle falls [28], and a lack of knowledge and skills is a commonly cited barrier experienced by healthcare staff, along with other factors, such as staffing levels and workloads [29]. LTCF staff surveys [30, 31] and interviews [32] concluded that staff in LTCFs focused more on extrinsic factors, such as environmental hazards, and that they had limited knowledge of intrinsic factors in terms of assessment and treatment.

We therefore performed an exploratory, sequential mixed-methods study, to understand falls prevention from the perspective of LTCF staff, so as to inform the development of effective LTCF staff training and education that could be widely implemented in practice. As part of this wider project, the key objectives of this study were to:

- Ascertain current staff practices for post-fall management, and current falls (prevention) care plans in their site
- Explore staff suggestions for fall and fall-related injury prevention in their site
- Identify staff suggestions for supporting the implementation of falls training into practice.

## Methods

We followed the guidelines of Strengthening the Reporting of Observational Studies in Epidemiology (STROBE) when reporting the current study [33, 34].

### Design

A self-administrated LTCF staff survey was used in this cross-sectional study. Results from the survey regarding staff knowledge attitudes and skills are published elsewhere [35]. The findings from six open-ended questions contained within the survey are presented in this paper. Two of the authors have extensive experience of residential care and the wider project was informed by health service staff with senior roles in residential care (a site education lead and a residential services manager).

### Setting and selection of participants

The setting was the counties of Cork and Kerry in southwest Ireland, which share governance and funding of residential care. In total, 71 LTCFs for older people were eligible (65 in Cork, six in Kerry). These LTCFs are all “nursing homes” with 24-h nurse availability, rather than “care homes”, and the vast majority of residents are over 65 and frail. Few (11%) have dedicated dementia units, although most residents in LTCF in Ireland have cognitive impairment [36]. A sampling framework was used to achieve variation based on three characteristics, i.e., provider type, facility size and location. Private providers are the most common type in Ireland, then public (state-funded and state-provided) units, while voluntary (not-for-profit) is rare. All provider types contain both single and multi-occupied rooms. The latter are common in public units (although reducing over time) but rare in private or voluntary units (e.g. a 60 bedded-unit might have 2 multi-occupancy rooms (2–3 residents) to accommodate a resident’s preference for company, but most are single rooms). Public sites may have 1 or 2 dedicated palliative care beds, but specialist palliative care input is similar to that provided to a person dying at home. Public and voluntary sites typically have some dedicated physiotherapy and occupational therapy hours; these services need to be purchased privately from a visiting therapist in private sites. General practitioners provide some hours to all sites. Facilities were stratified by size, into over 50 beds (the most common size) or under 50 beds (the least common), and by location (urban/rural; Cork/Kerry). Using a random sample generator, sites were selected within each sampling group to achieve a (20%) sample.

The study information was shared with selected sites by email and phone, by one of the research team. Sites that agreed to participate received formal invitation letters and promotional posters with links to the survey. LTCF staff were defined as those employed by the site, or with a contract to provide a private service to the site (e.g. where a single physiotherapist was available for private sessions in the site). This included the attending General Practitioner but not a visiting specialist team (e.g. geriatric outreach teams). Participant eligibility required at least three months of full-time or part-time work at the site, including all staff who provided direct care to residents (e.g., nurses, healthcare assistants, physiotherapists, occupational therapists, etc.). An invitation to participate was sent to all staff at a site that met the inclusion criteria, by the site gatekeeper/champion, as follows. A site champion, nominated by the director of nursing (DON), received invitation emails explaining the study and containing a link to the survey to share on social media and through staff email cascades. In some sites, the site champion preferred a paper version to facilitate staff preferences and avoid potential technical difficulties. Thus, 540 paper surveys were sent by post, along with postage-paid envelopes for returning the completed questionnaires within four weeks (2–3 envelopes per site, to allow return of each wave of responses). Two reminder emails were sent to champions, one and three weeks later, to encourage and remind potential participants. Site DONs identified medical officers or general practitioners (GPs), and they assisted in recruiting them. We provided them with an online link and/or invitation letter. Recruitment took place between April and August 2022.

### The survey instrument

This overall study survey included 38 questions. This composite survey blended two existing surveys as follow:

- The first one was the Fall Knowledge Test (Form 2E) from the Agency for Healthcare Research and Quality (AHRQ) falls toolkit [37]. This test was adapted by AHRQ from a self-assessment tool to test knowledge of a national nursing fall prevention clinical practice guideline in Singapore (for use in hospitals and LTCFs), where the knowledge tool is based directly on that guideline [38]. We used all 13 questions without any changes.
- The second survey (36 questions) explored staff knowledge, confidence and attitudes regarding fall prevention interventions [30, 31], developed using the framework COM-B (Capability, Opportunity, and Motivation to Undertake Health Behaviour Change). This survey had been based on previously validated surveys, and was reviewed for validity by its developers via a “talk through” pilot with five separate staff in LTCFs [29]. We excluded 14 questions that overlapped with the Form 2E questions, or were irrelevant to our study focus.
- We added three new questions: one demographic question (concerning job role) and two open-ended questions (regarding staff suggestions and comments on fall prevention activities).

Therefore, the final study survey (combining Form 2E and the adapted COM-B) comprised 31 closed-ended questions and seven open-ended questions (see Supplementary file 1).

The following items were explored via closed questions: i) demographic data (7 items) on age, gender, educational level, job role, years of experience working with older people, and in the particular LTCF, and shift work pattern; ii) knowledge-related questions (*n* = 13) with multiple correct answers, resulting in a possible total of 33 points; iii) self-rated attitudes and confidence items (*n* = 7) involved a five-point Likert scale; iv) previous fall prevention training and future educational preferences (*n* = 4). The latter was supplemented by an open-ended question on learning methods. These results are available [35].

The following open-ended questions form the basis of this paper, to address our research questions:

1. “List the top three things that could help your site to better prevent residents from falling”
2. “List the top three things that could help your site to better prevent residents from injuring themselves if they fall”
3. “What would you do if a resident had fallen during your shift?”
4. “Is there a falls prevention plan in the notes of the residents you are currently working with?”, followed by a request for details if positively answered
5. “What would help you use training you have received in practice to prevent falls when you are at work?”
6. Any other comments about falls prevention in residential care facilities?

Once built in Microsoft Forms, two nurses and one physiotherapist conducted pilot tests of the online survey to ensure the instructions were clear and that the question structure and wording were appropriate. Their feedback led to minor wording changes. They also estimated the completion time of the survey (10–15 min) to provide potential participants with this information.

### Data analysis

Using a single database, we combined online and paper-based responses. Within each site, the responses per site ranged from 1 to 25. In all site responses, we checked for duplication across seven demographic criteria, ranging from four to seven options per criterion. This duplicate check was performed using an Excel sheet. Content analysis was performed on the qualitative data from open-ended questions, applying both inductive and deductive approaches [39, 40]. We used NVivo software (QSR International) Version 2021 to organise the data for the coding and categorising process. Firstly, specific observations were classified into general statements/themes using an inductive approach based on the meaning of the words in the text, as follows. Open-ended questions were coded and categorised independently by two researchers (NA, CC); their findings were compared and discussed initially to resolve any conflict. Any remaining disagreements were resolved by a senior researcher (ST). Then, using a deductive approach, similar subcategories were classified using fall prevention domains from an Australian fall prevention guideline [21] separately for preventing falls and preventing fall-related injuries. We selected this guideline because it focuses primarily on residential care facilities (although dated- 2009), and is comprehensive. An existing Irish Falls Strategy was similarly dated (2008) but had little focus on LTCFs [41], and the *World Guidelines for Falls Prevention and Management for Older Adults* [10] was not published at the time of our study. In parallel, all subcategories were grouped according to the refined Theoretical Domains Framework (TDF). The TDF was created through a consensus-building process involving health psychologists and health service researchers to systematically analyse the behavioural change processes necessary to put evidence-based intervention into practice [42]. The refined TDF has 14 domains and 84 constructs [43].

The research team members discussed the results to promote reflection, and the final frequency of responses was calculated. We cross-tabulated the general (inductively generated) themes by job role to determine how LTCF staff approach fall prevention and fall-related injuries, as this would inform role-specific education. We combined DONs with senior nurses as “senior nurses”, as we expected that they would have similar levels of knowledge and clinical competences. All codes developed in this study are provided in the codebook (see Supplementary file 2).

### Ethics

The Social Research Ethics Committee at University College Cork (UCC) approved this study. All information collected in the survey was anonymous. The survey was voluntary; participants were informed about the study at the beginning and asked to tick a consent box if they were willing to participate. Only the researchers had access to hard-copy data, which was stored in locked cabinets. University hard drives were password-protected and used to store electronic survey data (when primarily electronic, or once transcribed from paper surveys).

## Results

### Participants’ characteristics

From 14 invited sites, 13 participated (93%), representing 18% of all sites in the region and employing a total of 1,039 staff. Six LTCFs were public; five were private; two were voluntary (50% of available voluntary sample), with nine large and four small sites. Two were in Kerry (33% of available sample), and seven were rurally based. The number of staff employed for under three months (and hence ineligible) is unknown; thus, the survey response rate was at least 15% (*n* = 155; range 1% to over 55% per site). Supplementary file 3 describes participant demographics. Most were female (*n* = 122, 78.7%), and aged 30–59 years (30–39 years: 27.7%; 40–49 years: 22.6%; 50–59 years: 23.2%).

Nursing staff were in a small majority, with 51 (32.9%) nurses and 32 (20.9%) senior nurses, the latter including seven DONs, followed by HCAs (*n* = 55; 35.5%). There were eight GPs, two physiotherapists, and also three administrators and two maintenance staff (grouped as "others"). Overall, 66 (42.6%) had experience working with older people for more than 11 years. Most respondents (*n* = 69) worked in their current LTCF for 3–24 months; 43 had been there for 11 years or more.

A bachelor's degree (European Qualification Framework level 6) was the most frequent educational level (*n* = 67, 44.4%; mostly nursing personnel). An education award at European Qualification Framework level 4/5 was attained by 40 participants (25.8%; predominantly HCAs). Most respondents worked full days (12 h), and most usually worked the same shift (e.g., always morning and afternoon, or morning only, or twilight hours only) (*n* = 72). Eight participants worked only night duty.

### Suggestions for preventing falls and fall-related injuries

Staff in LTCFs identified activities that they considered to be the “top three ways” to prevent falls (*n* = 129 responses) (Table 1) and fall-related injuries (*n* = 126 responses). These are presented below, mapped to the Australian Falls Guideline Categories (*Guideline categories* are *in italics*) [21]. Overall, e*nvironmental review and modification* was the most frequent suggestion for both fall prevention (*n* = 52; 40.31%) and for fall-related injuries (*n* = 74; 58.73%). The three most common environmental elements to prevent falls were environmental evaluation safety, clutter-free environments and non-slip flooring. Similar environmental elements were also considered to prevent fall-related injuries, with the additional frequent suggestion of low-level beds.

**Table 1:** Staff responses to the “top three items” to prevent residents from experiencing falls and fall-related injuries

Data from the “three top items that could prevent residents from experiencing falls”					Data from the “three top items that could prevent residents from having fall-related injuries”				
Sub-category detail and frequency		Assigned generic category and frequency	*N* = 129 (100% )	Corresponding main category in fall prevention guideline [24]	**Sub-category detail and frequency**		Generic category	*N* = 126 (100 %)	Corresponding main category in fall prevention guideline [24]
**Environmental safety evaluation**	26	Environmental design and safety	52 (40.31)	*Environmental review and modification*	**Clutter-free environment**	33	Environmental design and safety	74 (58.73)	*Environmental review and modification*
**Clutter-free environment**	18				**Environmental safety evaluation**	27			
**Non-slip Flooring**	12				**Low-bed levels**	19			
**Room layout/ building layout**	5				**Non-slip Flooring**	13			
**Appropriate lighting**	3				**Appropriate lighting**	7			
**Handrails/ side rails**	3				**Handrails/ side rails**	6			
**Locking furniture wheels**	2				**Appropriate Furniture**	4			
**Low-bed levels**	1				**Room layout/ building layout**	2			
**Appropriate Furniture**	1				**Crash mats**	28	Protective equipment	37 (29.36)	*Providing storage and equipment*
**Staff training /education**	30	Staff education/training/awareness	51 (39.53)	*Staff education*	**Appropriate mobility aids**	13			
**Staff Awareness**	15				**Provision of visual aids**	1	Sensory aids	1(0.79)	
**Ongoing education training**	6				**Hip protectors**			38 (30.16)	*Using hip protectors*
**Training targeting HCAs**	2				**Provision of sensor alarms**	17	Alarms/call systems	22 (17.46)	*Individual observation and surveillance*
**New staff induction training**	2				**Provision of call bells**	6			
**Staff skills (i.e., Appropriate Manual Techniques)**	2				**Resident supervision**	4	Observation approach (Monitoring residents)	7 (5.55)	
**Training delivered by PT/ OT**	1				**Hourly rounds**	4			
**Mandatory training**	1				**Residents Support from staff**	16	Resident care support	19 (15.08)	*Transfer and mobility assistance*
**Fall Audits**	1				**Rapid Response to needs**	3			
**Resident supervision**	28	Observation approach (Monitoring residents)	40 (31.31)	*Individual observation and surveillance*	**Bone health density and/or calcium and vitamin D intake**			15 (11.90)	*Vitamin D and calcium Supplementation/ osteoporosis management*
**Hourly rounds**	9								
**Regular Resident Toileting**	6								
**Resident Positioning close to nurses’ stations**	1				**Appropriate footwear**	13	Appropriate footwear	14 (11.11)	*Feet and footwear (i.e., safe footwear)*
					**Appropriate clothing**	3			
**Provision of sensor alarms**	19	Alarms/call systems	25 (19.38)		**Fall risk assessment**	8	Assessment of residents	13 (10.32)	*Fall risk screening and assessment*
**Provision of call bells**	8				**Post-fall assessment**	4			
**Identifying those at risk of falling (i.e., fall symbols)**	2	Flagging	2 (1.55)		**Assessment of residents (ongoing/Timely)**	1			
**Resident Support from staff**	17	Resident care support	21 (16.28)	*Transfer and mobility assistance*	**Exercise/ physical activity**	12	Exercise and physical wellbeing	11 (8.73)	*Exercise for preventing falls*
**Rapid Response to needs**	4				**Breathing exercise**	1			
**Fall risk assessment**	14	Assessment of residents	19 (14.73)	*Fall risk screening and assessment*	**Staff training /education**	5	Staff education/training/awareness	9 (7.14)	*Staff education*
**Assessment of residents (ongoing/Timely)**	4				**Staff awareness**	4			
**Pre/Post-admission assessment**	1				**Staff skills (i.e., Appropriate Manual Techniques)**	2			
**Appropriate mobility aids**	12	Protective equipment	17 (13.18)	*Providing storage and equipment*	**Resident Education/Awareness**	8	Resident Education	8 (6.35)	*Involving residents in fall prevention*
**Provision of essential items**	4								
**Provision of visual aids**	1	Sensory aids	1 (0.77)		**Medication review**			7(5.55)	*Reviewing medications*
**Medication review**			16(12.40)	*Reviewing medications*	**Staff knowing residents**			4 (3.17)	No corresponding category
**Exercise/ physical activity**	13	Exercise and physical wellbeing	14 (10.85)	*Exercise for preventing falls*	**Adequate staffing numbers**	2	Organisation factors that help to prevent falls	2(1.59)	No corresponding category
**Individualised exercise programme**	2								
**Walking Group**	1				**Staff communication**			1(0.79)	No corresponding category
**Resident Education/Awareness**	9	Resident and family education	11 (8.53)	*Involving residents and families in fall prevention*					
**Family Education/ Engagement**	5								
**Appropriate footwear**			6 (4.65)	*Feet and footwear (i.e., safe footwear)*					
**Adequate staffing numbers**	24	Organisation factors that help to prevent falls	33(25.58)	No corresponding category					
**Specialist care (Increased PT/OT input)**	11								
**Resident Group Size**	3								
**Staff Punishment**	2								
**Funding**	1								
**Staff knowing residents/ care plans**			12 (9.30)	No corresponding category					
**Staff communication**			9 (6.98)	No corresponding category					
**Staff self-efficacy in fall prevention**			1 (0.77)	No corresponding category					

*HCAs *Health care assistants, *PT* Physiotherapist, *OT* Occupational therapist

*Staff education* was the second most frequent suggestion for preventing falls (*n* = 51; 39.5%). Thirty respondents suggested general staff education/training, while 15 specifically mentioned awareness of fall risk factors. A few stated that staff training should be ongoing, targeted at new staff and skill-focused. Staff education was less commonly suggested for fall-related injuries: only nine focused on it. *Individual [resident] observation and surveillance* was the third most common suggestion for preventing falls. This included monitoring residents (n = 40; 31.31%), via a variety of approaches (e.g., direct supervision, hourly rounds, regular toileting) and devices such as alarms and call systems (*n* = 25; 19.38%).

For preventing fall-related injuries, after environmental review, the two most common suggestions focused on protection from falls, including *providing storage and equipment* (*n* = 38; 30.16%) (most suggested crash mats) and *using hip protectors* (*n* = 38; 30.16%). The fourth most common was *individual [resident] observation and surveillance*, focusing more on monitoring equipment (e.g., alarms) (*n* = 22; 17.46%) than staff supervision or regular rounding (*n* = 7; 5.55%).

*Providing storage* and *equipment* (*n* = 17), especially appropriate mobility aids (*n* = 12), was suggested for preventing falls, but not to the same extent as for fall-related injuries. *Fall risk screening and assessment*, *involving residents and families in fall prevention* and *exercise for fall prevention*, were similar for fall prevention and fall-related injuries, both suggested at much lower frequencies than environmental evaluation and modification. Sixteen respondents suggested *reviewing medications* for fall-related injuries, compared to seven who recommended it for preventing falls. *Feet and footwear* was mentioned more often for fall-related injuries (*n* = 16) than for falls (*n* = 6). From 126 respondents, 15 suggested *vitamin D and calcium supplementation* to prevent fall injuries. Some other suggestions could not be classified using the fall prevention guideline categories. Of these, 33 out of 129 respondents pointed to organisational aspects for preventing falls, with 22 indicating adequate staffing levels and 11 indicating the need for specialised care. Nine respondents indicated the value of ‘knowing residents’ for preventing falls, compared to four doing so for fall-related injuries.

### Staff suggestions for preventing falls, mapped to the (refined) TDF

**Fig. 1:**
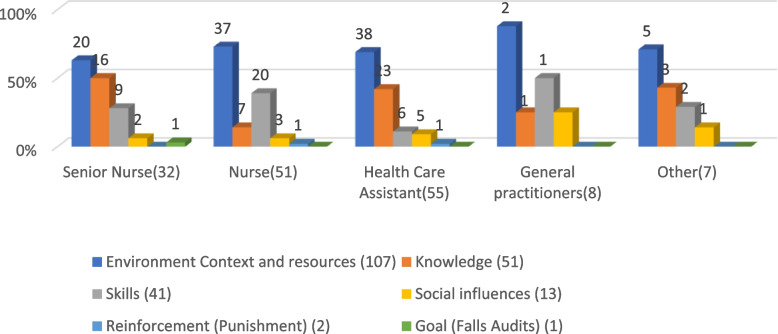
Mapping suggestions for fall prevention across job roles to TDF domains

Table 2 shows suggested fall prevention actions mapped to eight of 14 TDF domains (TDF domains are in bold text in the following section), while Fig. 1 gives the breakdown according to job role. Environmental context and resources was the TDF domain most commonly aligned to suggestions for preventing falls, with 201 suggestions from 107/129 respondents, representing nearly 2 suggestions per respondent overall, mapped to this domain (Table 2). Within this domain, environmental design and safety was overall the most common suggestion.

**Table 2:** LTCF staff responses, categorised by job role, for preventing residents from experiencing falls, mapped to the TDF (most common suggestion per job role are in bold)

**TDF Domain (Number of respondents)**	Categories/subcategories	Senior Nurse (28/32)	Nurse (43/51)	HCAs (45/55)	GP (7/8)	PT (2)	Other (3/5)	Total Suggestions (129/155)
**Environment Context and resources (*n* = 107)**	Environmental design and safety	9	**19**	18	3	**1**	**2**	52
	Monitoring residents (observation approach)	11	12	15	0	1	1	40
	Alarm and call systems	4	11	8	1	0	1	25
	Adequate staffing numbers	5	6	9	3	0	0	23
	Protective equipment	2	7	8	0	0	0	17
	Resident support from staff	4	7	4	2	0	0	17
	Specialist Care (Increased PT/OT input)	2	4	1	**4**	0	0	11
	Appropriate footwear	0	4	2	0	0	0	6
	Rapid Response to needs	0	1	3	0	0	0	4
	Resident Group Size	0	0	3	0	0	0	3
	Identifying those at risk of falling (i.e., fall symbols	0	1	1	0	0	0	2
	Funding	1	0	0	0	0	0	1
*Total domain suggestions*		*38*	*72*	*72*	*13*	*2*	*4*	*201*
**Knowledge (*n* = 51)**	Staff education, training, and awareness	**15**	6	**22**	2	1	1	47
	Resident and family education	4	2	3	0	1	1	11
*Total domain suggestions*		*19*	*8*	*25*	*2*	*2*	*2*	*58*
**Skills (*n* = 41)**	**Assessment of residents**	**4**	**10**	**2**	**1**	**0**	**1**	**18**
	Medication review	2	11	0	3	0	0	16
	Exercise and physical wellbeing	5	3	4	1	1	0	14
	Staff skills (i.e., Appropriate Manual Techniques)	1	0	1	0	0	0	2
*Total domain suggestions*		*12*	*24*	*7*	*5*	*1*	*1*	*50*
**Social influences (*n* = 13)**	Staff communication	2	2	2	2	0	1	9
	Rapid Response to needs	0	1	3	0	0	0	4
*Total domain suggestions*		*2*	*3*	*5*	*2*	*0*	*1*	*13*
**Reinforcement (*n* = 2)**	Punishment	0	1	1	0	0	0	2
*Total domain suggestions*		*0*	*1*	*1*	*0*	*0*	*0*	*2*
**Goals (*n* = 1)**	Falls Audits	1	0	0	0	0	0	1
*Total domain suggestions*		*1*	*0*	*0*	*0*	*0*	*0*	*1*
**Social- professional and identity/Belief of capabilities (*n* = 1)**	Staff self-efficacy in Fall Prevention							1
*Total domain suggestions*								1
Not mappable to TDF domain	Knowing residents	2	1	7	1	0	1	12
*Total domain suggestions*		*2*	*1*	*7*	*1*	*0*	*1*	12

*HCAs* Health care assistants, *PT* Physiotherapist, *GP* General practitioner

Most respondents suggested *environmental design and safety* and *monitoring residents*. Senior nurses suggested *resident monitoring* (11/32) and *environmental design and safety* (9/32) most often. The priorities of non-senior nurses, and the combined responses of PTs and ‘other groups’ were: *environmental safety and design* (19/51 and 3/7, respectively) and *resident monitoring* (11/32 and 2/7, respectively). The HCA’s suggestions mirrored others: *environmental design and safety* (18/55) and *monitoring residents* (15/55), with *adequate staffing levels* (9/55) suggested less frequently. Albeit with smaller numbers, GPs suggested *increased PT and OT input* (4/8), *environmental safety* (3/8) and *staffing levels* (3/8) (see Table 2).

Knowledge was the second most common TDF domain, aligned to *educating staff* and *educating residents and families*. This was suggested by all staff roles, including 42% (23/55) of HCAs and 50% (16/32) of senior nurses. Overall, *staff education* was suggested four times more frequently than *resident education*. Closely linked to knowledge, four categories of suggestion for preventing falls aligned to TDF skills (*assessment of residents, medication review, exercise and skills acquisition*), which were suggested especially by nurses (20/51, 39%) and GPs (4/8, 50%). Almost all staff, especially nurses, suggested *resident assessment* and *medication reviews*. All except the “others” emphasised *exercise* (noting the small number of respondents in this group).

*Staff communication* and *rapid response to residents' needs* were suggested by a few respondents, across all job roles, which mapped to the TDF social influence domain. *Rapid response to needs* was also mapped to the TDF domain environmental context and resources. Three HCAs stated that responding to residents' needs on time was very important.

Table 2 details other fall prevention priorities, albeit only from a few staff members, which mapped to four additional TDF domains. A *falls audit*, suggested by one staff member, corresponded to the TDF goals domain, while [*staff*] *punishment*, as suggested by two staff members (defined in the TDF as a ‘painful, unwanted or undesired event or circumstance imposed as a penalty on a wrongdoer’; but in healthcare typically being a penalty such as some reduction in work autonomy privileges, a probation period, or in extreme events dismissal), was mapped to the TDF domain of reinforcement. An inductive category entitled *staff self-efficacy in fall prevention* was mapped to two TDF domains: social-professional and identity, and belief in capabilities (responses referred to both).

Twelve staff members (including seven HCAs) prioritised *getting to know the residents and their care plans*, which did not correspond to a TDF domain.

### Staff suggestions for preventing fall-related injuries, mapped to the (refined) TDF

**Fig. 2:**
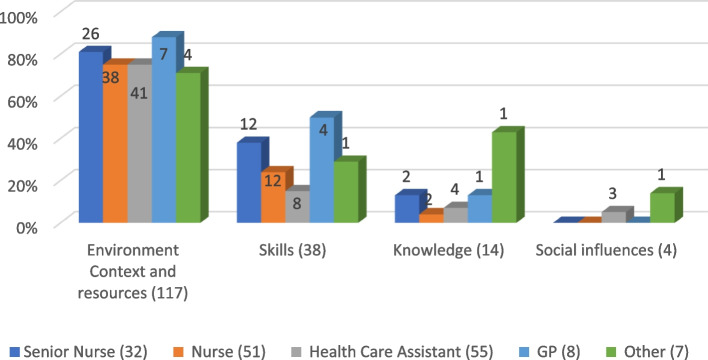
Mapping suggestions for fall injury prevention across job roles to TDF domains

Suggestions for preventing fall-related injuries were mapped to only four of the 14 TDF domains (Table 3); the variation in suggestions across job roles is summarised graphically in Fig. 2. Similar to its predominance regarding fall prevention, in total there were 214 suggestions, from 117/126 respondents, mapping to the TDF domain of environmental resources and context. The five most common suggestions for preventing fall-related injuries mapped to this domain, and were *environmental design and safety* (the predominant category), *protective equipment*, *hip protectors*, *providing alarms/call system* and *resident support from staff* (Table 3). Apart from physiotherapists and GPs, providing *protective equipment* was the second most frequent solution; this was also a more frequent suggestion for preventing injuries than for preventing falls. Closely linked to this theme, *hip protectors* were the third most common solution. GPs most commonly recommended *environmental safety and design* (6/8), followed by *hip protectors* (4/8).

**Table 3:** LTCF staff responses, categorised by job role, concerning actions to prevent residents from experiencing fall-related injuries, mapped to the TDF (most common suggestion per job role *in bold)*

TDF Domain (Number of respondents)	Categories/subcategories	Senior Nurse (29/32)	Nurse (41/51)	Health Care Assistant (42/55)	GP (7/8)	PT (2)	Other (4/5)	Total (126/155)
Environment Context and resources (*n* = 117)	Environmental design and safety	**21**	**24**	**19**	**6**	1	**3**	74
	Protective equipment	8	16	11	0	0	3	38
	Hip protectors	8	9	14	4	0	3	38
	Alarm and call systems	4	5	12	0	1	0	22
	Resident support from staff	1	6	8	0	0	1	16
	Appropriate footwear	3	4	7	0	0	0	14
	Monitor residents	0	2	4	0	0	1	7
	Rapid response to needs	0	0	3	0	0	0	3
	Adequate staffing numbers	0	1	0	1	0	0	2
*Total domain suggestions*		*45*	*67*	*78*	*11*	*2*	*11*	*214*
Knowledge (*n* = 14)	Staff education and awareness	2	1	3	0	1	1	8
	Resident education	2	1	1	1	**2**	0	7
*Total domain suggestions*		*4*	*2*	*4*	*1*	***3***	*1*	*15*
Skills (*n* = 38)	Assessing bone health density and/or calcium and vitamin D intake	6	5	1	3	0	0	15
	Assessment of residents	3	6	4	0	0	0	13
	Exercise and physical wellbeing	3	2	3	1	1	0	10
	Medication review	2	1	1	2	0	1	7
	Staff skills (i.e., Appropriate Manual Techniques)	0	1	0	0	0	1	2
*Total domain suggestions*		14	15	9	6	1	2	*47*
Social influences (n = 4)	Rapid response to needs	0	0	3	0	0	0	3
	Staff communication	0	0	0	0	0	1	1
*Total domain suggestions*		0	0	3	0	0	1	*4*
Not mappable to TDF domain	Knowing of residents	0	0	3	0	0	1	4
*Total domain suggestions*		0	0	3	0	0	1	4

*HCAs* Health care assistants, *PT* Physiotherapist, *GP* General practitioner

Mapping to the TDF knowledge domain, all groups except GPs recommended *staff education* for falls injury prevention. *Education for staff* and *residents* were suggested approximately equally for falls-related injury prevention, although both at a lower frequency than for falls prevention. Solutions that mapped to the TDF skill domain were most frequently suggested by senior nurses (12/32; 37.5%), followed by GPs (4/8). Within this domain, *assessing bone density and/or calcium and vitamin D intake* was the most common, suggested by senior nurses (6/32), nurses (5/51) and GPs (3/8), being the sixth most frequent category for falls-related injury prevention. *Assessment of residents*, *exercise* and *physical wellbeing* were the other common responses mapped to the skills domain.

The TDF domain of social influences appeared less relevant for preventing fall injuries than for preventing falls. Four staff focused on *getting to know residents* as a way to reduce fall injuries, which was not mapped to a TDF domain.

### The current practice of LTCF staff when responding to a resident’s fall during their shift

From the 155 participants, 131 detailed current practice when a resident falls (Table 4). We matched all inductively derived categories/subcategories with the Australian fall prevention guideline categories [21] (in italics below), except "protect[ing] residents’ dignity", which did not fit with any item or theme within the guideline.

**Table 4:** Care staff responses regarding actions if residents had fallen during their shift

Fall prevention Australian Guideline categories		Category (from inductive coding)		(Inductive) sub-category		Senior Nurse (29/32)	Nurse (37/51)	HCAs (51/55)	PT (2)	GP (5/8)	Other (5)	Total (131/155)
**Post-fall assessment (managing residents immediately after falls)**	**Check for injuries**	Resident assessment after falling		Assessment for injuries		**24**	**30**	13	2	2	1	**72**
	**Take baseline measurements**			Neurological assessment		10	19	2	0	0	0	31
				Vital signs assessment		7	8	4	1	1	0	21
				Post-fall assessment (unspecified)		8	12	4	0	0	2	26
				Pain assessment and management		4	4	1	1	0	1	11
	**Comprehensive assessment of falls**			Fall risk assessment		10	8	1	0	3	0	22
				Fall circumstances assessment		2	2	2	0	0	0	6
**Responding to fall incidents**		Care staff initial responses to falls amongst residents		Call for help		6	9	**34**	**2**	**2**	2	56
				Call staff nurses for residents’ examination		1	0	33	1	0	4	39
				Do not move residents		1	4	14	1	0	1	21
				Call an ambulance for transfer to hospital if needed		7	6	4	2	0	0	19
				First aid		4	2	1	0	0	0	7
**Move the resident**		Ensuring the safety of residents after falling		Safe transfer	Use of Hoist	10	3	14	1	0	1	29
					Safe transfer of residents by staff	6	6	4	0	0	1	17
				Assessing safety for transfer and mobility		1	4	10	1	0	0	16
				Ensure the surrounding environment is safe		2	6	5	0	0	0	13
				Follow nurse instructions Or Policy		0	1	10	1	0	0	12
**Monitor the resident**		Monitor Residents		Monitor Resident		3	6	2	2	1	1	15
**Reporting and recording falls**		Documenting falls in residents’ records;		Complete fall report		11	17	9	0	1	0	38
				Update care plan		5	7	0	0	1	0	13
**Communicate to all relevant staff, family, and carers**		Informing MDT members/families of residents	Informing MDT members	Inform Doctors or GPs		16	16	4	1	1	0	38
				Inform the authorised staff (i.e., the senior nurse, manager, ADMIN, DON)		2	7	4	0	1	0	14
				Refer to physio		5	4	0	1	0	0	10
				Inform all staff		2	1	0	0	0	0	3
			Informing families of residents	Inform family OR relatives		14	10	1	0	1	0	26
**Discussing and analysing the fall and future risk management**		MDT fall analysis		MDT fall analysis		2	1	0	0	1	0	4
**Reassure and comfort the resident**		Ensuring resident comfort and calmness		Keep resident comfortable		5	9	13	0	2	1	30
				Reassure Residents		7	3	11	0	0	1	22
**Not mappable to guideline**		Protect the residents’ dignity		Protect the residents’ dignity		0	0	2	0	0	0	2

*HCAs* *Health* care assistants, *PT* Physiotherapist, *GP* General practitioner

*Resident assessment post-fall:* The most frequently cited response overall (72/131 responses), and the most common response for nurses and senior nurses, was that respondents check for injuries. Many respondents (*n* = 26) did not specify what this involved (e.g., “a post-fall assessment”), while a few mentioned standardised approaches (e.g., a “two-minute rule” assessment, head-to-toe examination, full body assessment). As expected, senior nurses and nurses were more likely than HCAs to report taking objective measurements after falling, including specific measurements such as neurological observation, vital sign checks and others. Nurses and senior nurses reported conducting fall risk assessments more often than HCAs, evaluating fall risk factors and/or fall circumstances.

*Responding to falling incidents:* Calling for help was the most common response aligned to this guideline category (*n* = 56/131), as reported particularly by HCAs (34/51) after a fall incident. Following that, the need for nurses to examine fallen residents and to avoid moving him/her from the floor, were commonly reported particularly by HCAs. Senior nurses more often reported calling an ambulance to transfer residents to a hospital if necessary and providing first aid.

*Move the residents:* Twenty-nine respondents, mostly HCAs and senior nurses (14 and 10, respectively) reported using hoists to transfer residents from the floor; 17 reported that residents were transferred by staff. In total, 16 respondents, of whom 10 were HCAs, reported performing an assessment of the faller's ability to transfer him/herself safely to a chair or bed. Of the 13 respondents who re-checked environmental safety before transferring fallers, six were nurses, and five were HCAs. HCAs reported following the fall policy or nurses' instructions. *Monitor the residents:* A total of 15/131 respondents from all job roles indicated supervising residents after falling.

*Reporting and recording falls:* Only 38 of the 131 respondents reported completing a fall incident report, while 13 reported updating the care plan. There was no mention of fall documentation by HCAs, physiotherapists or “others”. *Communicating with all staff members, families and carers:* According to 38/131 respondents, GPs should be informed about fall incidents (16/37 nurses and 16/29 senior nurses reported this). Furthermore, 26 indicated the importance of informing residents' families, including 14/29 senior nurses and 10/37 nurses. Only a few (10/131) respondents, including five senior nurses and four nurses, mentioned the value of referring patients to physiotherapists. *Discussing and analysing falls and future risk management:* Only four respondents reported holding MDT meetings to discuss and analyse cases of falls.

*Reassure and comfort the resident:* 30 respondents (including 13/51 HCAs and 9/37 nurses) reported maintaining the comfort of fallers in their assumed position (e.g., a pillow or blanket), and 22 (11/51 HCAs and 7/29 senior nurses) referred to reassuring residents. Two HCAs reported protecting residents’ dignity, which was not mapped to a guideline category but demonstrated a caring approach.

### Fall prevention care plans

Most respondents (72.9% (*n* = 113)) reported fall prevention care plans being in place for residents, and 70 provided details of a typical fall prevention care plan. Approximately half detailed equipment provision (38/70) and environmental and transfer safety issues (32/70). Assessment of fall risks (27/70) and resident monitoring, instructions and documentation (20/70) were detailed less frequently. Only 8/70 reported resident-centred strategies such as motivating residents.

### Fall prevention training – value, content and implementation of training into practice

Participants were asked what would help them adhere to fall prevention training in their work practice (*n* = 79 responses), before a final “any other comments” question (*n* = 27 responses). These data are presented together, as there was much overlap in responses; many discussed the training itself rather than its implementation (see Supplementary file 4).

Overall, 54 respondents indicated the importance of additional staff education in fall prevention in their workplace. Twelve recognised its value for their personal knowledge and skills, e.g., “*The training would ensure that I am better able to identify the risk of falls to ensure that residents are equipped with aids that would prevent falls”.* Five suggested the training should be compulsory for all, with four suggesting it as part of staff induction: *“All staff to have the same level of education in falls prevention”*. Two indicated the importance of staff teamwork and a multidisciplinary approach to training: *“all staff to have fall knowledge through training so as to successfully work as a team”*. Three mentioned the importance of training for staff motivation and self-efficacy. Conversely, another indicated that staff motivation was intrinsic: “*No training is going to ensure that people will do the right thing. It has to come from the person within”*.

Suggested methods of delivery included in-service training, e.g., *“On-site practical training following education is beneficial”* or one-on-one training provided by an external trainer, e.g. *"We would greatly appreciate in-house training from others outside our facility".* Eleven highlighted the necessity of manual handling training to safely manage a fall incident. Four suggested specific educational resources (i.e., videos, leaflets, lectures and posters). A few highlighted the importance of training being understandable and raising staff awareness of fall risk factors; four suggested training should be “appropriate”.

The most common suggestion to improve the implementation of training in practice included staff communication and peer learning (*n* = 12), such as *“Interacting with staff and asking questions and asking how I can improve”* and “*The knowledge to educate other staff confidently about what I learnt and what possible improvements can help in preventing falls”*. Other suggestions included fall audits and feedback (7/26), reminders for staff (2/26) and fall champions (2/26) (see Supplementary file 4).

*LTCF staffing issues:* Staffing issues was commonly reported; 13 indicated challenges with staffing levels, e.g., “*One cannot ignore the ongoing resource issues that influence falls, particularly resident-staff ratios* and “*A huge divide between what the HSE homes are provided with versus the private/voluntary homes […] the staff-to-resident ratio is not the same in non-HSE homes: it is varied and diverse.* The need for consistency in staff in a given ward was important, e.g., “*Stop moving staff between wards every single day*”. There was an indication of needing specialist input such as that of PTs, OTs and maintenance staff (see Supplementary file 4). One respondent stated that residents require one-to-one care to prevent falls: *“It is difficult, and the only way to prevent falls 100% is to provide 1:1 care to fall-risk residents, which is not possible*”.

*Involvement of residents in fall prevention:* Few recommended involving residents in fall prevention; this was in relation to increasing awareness levels (*n* = 2), having staff and residents communicate (*n* = 1), and involving residents in practice (*n* = 1). Four perceived that fall prevention (i.e., over-monitoring) increased fear of falling and frustration, reducing autonomy, e.g., *“We reduce people’s autonomy to make choices and end up curtailing their movement for fear of a fall. Life is a risk! Physical restraints may be eliminated, but constant monitoring must be very frustrating for our residents*”; and “*We must take risks and let frail residents mobilise, but litigation is now becoming an issue in Irish healthcare”*. Three indicated the significance of reducing fear of falling by improving residents’ mobility, and two indicated improving residents’ physical activity: *“Falls cannot be eliminated while promoting quality of life. Residents must not be confined by fear of falling, and should be encouraged with mobility”*; *and* “*There is inadequate activation of residents […] Any training in fall prevention should include encouragement to get residents moving”*. Only two respondents mentioned educating families.

## Discussion

Healthcare professionals in LTCFs are responsible for the care of residents who are intrinsically at high risk of recurrent falling. Implementing evidence-based recommendations requires them to understand the factors that cause falls, as well as how they can be treated and managed. Our study explored staff suggestions for preventing falls and fall-related injuries, along with current responses to fall incidents, and fall (prevention) care plans. A key finding is that LTCF staff felt a need to enhance the implementation of fall prevention activities in their sites alongside existing fall prevention efforts. LTCF staff across all disciplines were focused most on preventing falls and fall-related injuries by addressing extrinsic risk factors, corresponding to the “environmental review and modification” category from the fall prevention guideline and the TDF domain of “environmental context and resource”. This is aligned with previous survey- and interview-based studies where LTCF staff were more concerned with extrinsic fall risk factors (i.e., preventing environmental hazards) than intrinsic factors [30–32]. Multifactorial interventions have been shown to be effective in many SRs, as environmental reviews and modifications were incorporated [4, 17, 18]. Information regarding the effectiveness of environmental reviews and modification, as a single intervention, or on interventions tackling specific environmental hazards (e.g. low bed levels), however, has been lacking.

Resident observation and surveillance were commonly suggested to prevent falls. Using alarms/call systems was primarily recommended by nurses and HCAs for preventing falls and fall-related injuries. As outlined in a 2019 systematic review (SR), alarm devices alone have no effect- they must be part of a comprehensive care plan, and they can cause staff burden. In a Cochrane review, only one trial tested a wireless position-monitoring device (a skin patch on the thigh), and it found no evidence of a fall reduction [4]. As a single intervention, there were a lack interventional trials that evaluated the effectiveness of assistive devices, such as bed-exit alarms. Equally, direct monitoring by staff of residents at high risk of falls would allow the identification of behaviours that are risks for falls, as suggested by all staff in our study except GPs. In a previous study in the US involving focus groups with 55 nurses and 22 HCAs [44], monitoring of residents by staff who were experienced in falls was identified as one of the keys to preventing falls. A multi-site qualitative study across seven LTCFs in Canada (*n* = 98 LTCF staff) found that monitoring and supervision were perceived to be the most effective ways of preventing falls [28]. However, staff in our study paid less attention to monitoring residents after fall incidents; and monitoring was less commonly suggested for preventing fall-related injuries than for preventing falls. Our findings align with surveys conducted in Australia across 8 LTCFs, where only five of 147 staff considered resident observation valuable for preventing fall-related injuries [31]; mirroring a previous survey at a single site by the same group [30]. These differences suggest possible cultural differences across countries, and that this area needs to be included within educational programmes, bearing in mind the staff resources required in directly monitoring residents which might affect implementation in practice.

Our research identified that adequate staffing is perceived to be key for fall prevention. Staffing levels must be considered when determining staff members’ ability to provide fall prevention interventions [44] and the impact of staffing levels on residents’ safety [45]. A previous study used direct practice observation, interviews with residents and staff (*n* = 118), and chart abstraction across 21 LTCFs in the US to measure the level of care provided by nursing assistants [46]. This study found that nursing homes with higher levels of staffing provided better toileting assistance, repositioning and walking exercises, compared to those with low staffing levels. In another study, nurses in 112 nursing homes in the US shared their eight-year experience with implementing fall prevention programmes over four phases. Inadequate staffing affected other responsibilities and the ability to provide direct care to prevent falls, such as monitoring residents and providing toileting assistance [47].

Hip protectors are probably effective for preventing hip fractures from falls in LTCFs where staff are available to provide donning/doffing support [48], although even in this setting, there are challenges regarding compliance [49, 50]. Nursing education about the value of hip protectors reduced the number of hip fractures caused by falling in a cluster-randomised trial [51]. Our findings show that nursing staff in LTCFs in Ireland already believe hip protectors to be important for preventing fall-related injuries [52]. Furthermore, our results demonstrate that HCAs and nursing staff also believe in using a crash mat as protective equipment to reduce fall-related injuries, as aligned with the findings of William et al. [28]. The use of floor mats has been shown to reduce the risk of head and pelvic injuries for all drop heights [53]. However, they may pose a risk to those who are ambulatory and who have gait impairments or who use assistive devices to walk [54].

A concerning finding was that limited attention was given to preventive strategies focused on resident-related risk factors in our findings. Multifactorial falls risk assessments are the first step in many fall prevention guidelines for identifying any potential risk factors in an individual that increase falling [1, 20, 21, 38]. According to our results, LTCF staff placed a lower priority on assessing residents for risks of falls or fall-related injuries, or for post-fall management of risks. Nurses placed the greatest value in assessing residents compared to other job roles, as they play a vital role in fall assessment [22, 23]. A previous small qualitative study, unsurprisingly, had found that HCAs are less knowledgeable than nurses about biomedicine-related falls [32]. Consequently, fall risk assessment should be viewed as a primary method of preventing falls, requiring critical thinking skills and staff knowledge [55], while HCA training should include some training on intrinsic risk factors.

Medication review is another important fall prevention activity, since polypharmacy and high-risk medications (e.g., diuretics, benzodiazepines, and antipsychotics) increase the falls rate in LTCFs [3, 14, 56]. A previous qualitative study reported that nursing home staff should receive more training on the association between medication and falls [44] and that educational interventions on medication may reduce the risk of falls [57]. Vitamin D supplementation was found to be an effective single intervention for lowering the fall rate in LTCF residents in a Cochrane review [4], given the frequency of deficiency in this population. Our findings showed, however, that few respondents prioritised vitamin D for preventing fall-related injuries. This is consistent with the previous Australian multi-site survey, where only 5/147 staff across eight LTCFs suggested it as a prevention intervention [31].

Muscle strength, balance, coordination and bone health are improved by exercise, and fall prevention requires LTCF residents to exercise for 35 to 45 min twice per week [58]. Our findings demonstrate that few staff members focused on the benefits of exercise and improving physiotherapy input to prevent falls. Similarly, a small qualitative study conducted in Norway nurses and HCAs were unaware of the importance of exercise in LTCFs, as their main focus was protecting residents, rather than improving their independence and mobility [32].

Fall prevention requires a team effort, with all disciplines taking responsibility [22]. Multifaceted strategies, including staff communication, audits/feedback, fall champions, reminders and identification systems, have effectively increased the use of risk assessment and staff knowledge of fall prevention activities in other settings [59]. Our recent SR identified many fall prevention strategies used in LTCFs that had no evidence of effectiveness [60]. Similarly, in our survey, a few participants commented on mindfulness approaches in staff to help prevent falls. By communicating preventative measures and health promotion with professionals and residents, targeted interventions can be carried out to reduce falls [23, 61]. Future studies should examine the impact of these critical intervention implementation strategies on fall management.

It is noteworthy that this study emphasises the perceived importance of staff education in LTCFs as a significant factor for preventing falls. According to a recent SR from 2020, staff education interventions can reduce the numbers of falls and reoccurrences among residents [17]. Our survey responses indicated several important elements for future education programmes as suggested by respondents (e.g., in-service training and including manual handling as a topic) and as evidenced by less focus despite effectiveness (e.g., resident assessment, vitamin D, exercise, etc.).

Interestingly, the results showed that staff use quite a standardised approach when responding to falling incidents, with responses indicating knowledge of holistic and intensive post-fall control strategies. The World Fall Guideline advocates ‘post-fall assessment’, to determine the cause of the fall and any injuries that may have resulted, as well as re-reviewing the resident's fall risk factors, altering the intervention approach for the resident, and preventing needless hospital transfers [10]. As appropriate to their roles, HCAs focus more on responding to fall incidents and transferring residents safely, whereas senior nurses and nurses focus more on post-fall assessment, as has been reported by others [44]. However, there was less of a focus on reporting falls and updating care plans in staff apart from nurses in our study. In hospitals, completing post-fall documentation has been shown to increase staff awareness about fall risks and preventing falls [62]. Accurate fall reporting also makes audits and feedback more comprehensive.

When residents fell, most senior nurses involved family members by informing them regarding the incident. Family involvement may increase healthcare efficiency, effectiveness and the health of the population [63]. However, few respondents mentioned fall prevention training and educating residents, and few discussed how important it is to support the well-being and dignity of residents during fall incidents, along with considering their independence and freedom. These findings are consistent with the previous qualitative study which found that nursing staff in LTCFs were focusing more on fall prevention and protection than safety promotion, thus demonstrating a lack of person-centred care approaches, potentially compromising the dignity and well-being of residents [32]. Nurses should focus on communicating with residents and their families, along with improving resident education, as part of a holistic approach to fall prevention.

### Implications for future research and clinical practice

- The evidence base for the effectiveness of interventions targeting falls external risk factors needs to be strengthened, with additional research, especially randomised controlled trials. Environmental hazards and modifications (e.g., the lowering of beds level), alarm sensors, and crash mats have rarely been studied as individual interventions in LTCFs. Future studies need to examine these as individualised interventions, or have sufficient power to be able to adjust for other intervention effects.
- Across all LTCF staff roles, education and training aimed at improving their knowledge of intrinsic risk factors is necessary. The benefits of interventions such as exercise, medication review and vitamin D supplementation for preventing falls and fall-related injuries should be incorporated into future educational programmes for LTCF staff.
- The role of a fall risk assessment as a primary method of preventing falls needs to be included in all staff education programmes and fall prevention policies.
- Falls prevention needs to include residents and families as much as possible.

### Limitations and strengths

The study collected extensive information about what LCTF staff feel are the most important elements of fall and fall injury prevention, and their current practices. Various LTCF provider types and sizes, both urban and rural, were included, and 13 out of 14 invited sites participated, with an overall good sample size. However, despite the study having targeted all staff in LTCFs, GPs and health and social care professionals were underrepresented among the respondents. Although we offered online and paper versions of the survey, response rates were low, and variable across sites. LTCFs may have been affected by the COVID-19 pandemic through altered work practices and workload increases, along with LTCF staff not having protected time to participate in such surveys. Furthermore, site management, champion involvement and site culture may have influenced response rates, as indicated by the notable differences in response rates across sites (ranging from 1 to 55%). Low response rates in a site can indicate possible response bias, where those with good knowledge and interest in fall prevention are more likely to respond. All job roles were included in this study, resulting in enhanced validity, supported further by including respondent quotes. Data credibility was increased via the analysis of the data by two independent researchers. In this study, we mapped the data to both the refined TDF framework and an existing fall prevention guideline, which allowed us to identify new directions for improving fall prevention in LTCFs.

## Conclusion

LTCF employees, across all disciplines, placed a great emphasis on environmental context, resources/environmental review and modification, along with staff education, monitoring residents and using alarms/calling systems, for preventing falls and fall-related injuries, and on protective equipment and hip protectors for preventing fall injuries. Staff used a standardised, comprehensive approach when responding to falls. Some activities, such as assessing residents, providing exercise, reviewing medication and supplementing vitamin D, appeared under-recognised. Educating LTCF staff on fall prevention should take into account key context factors such as nursing home culture, their specific knowledge gaps and self-identified learning needs so that they can fully incorporate fall prevention activities into their practice.

### Supplementary Information

**Additional file 1:** **Supplementary file 1.** The survey instrument.

**Additional file 2:** **Supplementary file 2.** Data codes and their descriptions of open-ended questions.

**Additional file 3:** **Supplementary file 3.** Participant demographics.

**Additional file 4:** **Supplementary file 4.** Fall prevention training – value, content and implementation of training into practice.

## Data Availability

All datasets used and analysed during this study is available in from the corresponding author on a reasonable project.

## Abbreviations

LTCF: Long-term care facilities
TDF: Theoretical domains framework
HCAs: Health care assistants
STROBE: The strengthening the reporting of observational studies in epidemiology guidelines
COM-B: The capability, opportunity, and motivation to undertake a health behaviour change framework
AHRQ: The agency for healthcare RESEARCH and quality
MDT: A multidisciplinary team
DON: The director of nursing
GPs: General practitioners
PTs: Physiotherapists
OT: Occupational therapist
SREC: The social research ethics committee
UCC: University college cork
SR: Systematic review
HSE: The health service executive
