# Supplementary material for: Staff’s insights into fall prevention solutions in long-term care facilities: a cross-sectional study
Source: BMC Geriatr. 2023 Nov 13;23:738. doi: 10.1186/s12877-023-04435-7 (PMC10644547; doi:10.1186/s12877-023-04435-7)
Supplement: Supplementary file 2 — Additional file 2: Supplementary file 2. Data codes and their descriptions of open-ended questions. [file 12877_2023_4435_MOESM2_ESM.docx]

Supplementary file 2: Data codes and their descriptions of open-ended questions

| Code | Description |
| --- | --- |
| Non-slip floors | Refers to dry floors, safer contact with floors that have uneven surfaces, proper flooring and flooring types (e.g., stone) |
| Residents’ room/building layout | Refers to layout, more space and better ergonomics |
| Clutter-free environments | Refers to de-cluttering the environment, removing obstacles, cleaning the walking pathway and creating a safe walking space |
| Locking furniture wheels | Refers to the locking of any equipment or furniture, such as beds, tables, wheelchairs and so on |
| Provision of sensor alarms | Refers to the sensor alarms of any furniture or equipment, such as mats, beds and chairs, as well as a motion sensor |
| Appropriate mobility aids | Refers to walking aids, assistive devices) and equipment for standing (e.g., Zimmer frames; rollators) |
| Provision of essential items | Refers to the provision of necessary/proper items, equipment and supplies which are easy for residents to reach |
| Provision of handrails/side rails | Refers to the provision of bed rails, handrails and side rails |
| Hourly rounds | Refers to regular checks, regular rounds, hourly rounds, frequent checks and intentional rounds |
| Resident supervision | Refers to frequent observation, monitoring and supervision of residents |
| Resident support from staff | Refers to assisting residents with daily living activities, transfer, mobility, standing, walking and assistance in general provided by staff, along with identifying residents’ care needs |
| Exercise/Physical wellbeing | Refers to regular exercise, balance and weight bearing, breathing, strengthening the physio regimen and daily activities (walking, mobility physical activity) |
| Environmental safety evaluation | Refers to evaluating/checking environmental safety and removing hazardous and harmful things such as sharp objects |
| Provision of call bells | Refers to the provision of a call bell near residents |
| Appropriate furniture | Refers to comfortable furniture (e.g., beds, chairs), pillows and cushions |
| Hip protectors | Refers to hip protectors, hipsters, hip stoles and corner protectors |
| Crash mats | Refers to crash mats and the provision of mattresses and soft mats |
| Assessment of injuries | Refers to injury assessments related to limbs, head to toes, the body as a whole, head injuries, fractures and dislocations, along with full screening/assessment from head to toe, the two-minute rule, minor or major injuries, range of motion assessment and skin assessment |
| Medication review | Refers to regular medication review, sedatives used and bisphosphonate used |
| Bone health density/Calcium and vitamin D intake | Refers to bone health, vitamin D and calcium supplementation, the management of osteoporosis and anti-porosis needs |
| Staff knowledge of residents/care plan | Refers to knowing the resident's history (i.e., falls, medical conditions) and knowing the fall care plan, reviewing and checking the general care plan, the speciality care plan of PT and reviewing the care plan on a regular basis |
| Resident-centred strategies | Refers to resident-focused care, identifying and understanding residents’ needs, motivating residents and involving them in their care plan |
| Staff awareness | Refers to awareness of high levels of risk, the consequences of falling, infection, vigilance and alertness |
| Rapid response to needs | Refers to promoting responses via call bells or alarm sensors and reacting swiftly to residents when necessary |
| Use of hoist | Refers to the use of a hoist lift to transfer residents back to the bed, chair, room, and so on |
| Call for help | Refers to calling for help, calling for assistance and ringing the emergency bell to notify staff |
| Provision of visual aids | Refers to providing visual aids to residents, such as glasses |
| Resident education/awareness | Refers to educating residents on how to fall and risk factors, as well as increasing their awareness of the risk factors of falling |
| Family education/engagement | Refers to involving family members and educating them about falls, including how to prevent them |
| Appropriate lighting | Refers to providing proper, adequate lighting |
| Adequate staffing numbers | Refers to adequate allocation of staff, more staff needed |
| Low-bed levels | Refers to lowering the levels of beds |
| Staff training/education | Refers to educating/training staff in relation to falls prevention |
| Ongoing education and training | Refers to regularly training/educating staff about falls risks and fall prevention |
| Training targeting HCAs | Refers to educational training targeted at health care assistants |
| New staff induction training | Refers to educational training directed at all new employees |
| Staff skills | Refers to skills introduced during staff training (e.g., manual handling) |
| Mandatory training | Refers to mandatory staff training |
| Training delivered by PT/OT | Refers to a physiotherapist or occupational therapist providing training |
| Regular resident toileting | Refers to addressing residents’ toileting needs on a frequent basis |
| Resident positioning close to nurses’ stations | Refers to ensuring that residents are located near nurse stations |
| Identifying those at high risk of falling | Refers to the use of fall symbols or patient identifiers for residents at high risk of falling |
| Fall audits | Refers to conducting fall audits to promote fall prevention |
| Fall risk assessment | Refers to assessing the residents, identifying risk factors, quick screening, continuous assessment, identifying fall scores and multifactorial assessment, along with the FRASE scale, admission assessment and fall prevention checklists |
| Assessment of residents (timely/ongoing) | Refers to the process of assessing residents frequently and on a timely basis |
| Pre/post-admission assessment | Refers to the assessment of residents at the site prior to and after admission |
| Specialist care | Refers to providing specialty care such as physiotherapy, occupational therapy and maintenance |
| Resident group size | Refers to reducing the crowding of residents |
| Punishment | Refers to sanctioning or dismissing staff members who have made mistakes or having a staffing system which highlights anything untoward happened |
| Funding | Refers to the cost of equipment and required resources |
| Staff communication | Refers to staff communication, team communication, staff handover, staff discussion and multidisciplinary discussion |
| Staff proficiency in fall prevention | Refers to having ownership for resolving fall issues instead of waiting for others to do so |
| Appropriate footwear | Refers to having shoes that fit properly, non-slip shoes, proper footwear and proper clothing. |
| Post-fall assessment | Refers to assessing residents after falling or incidents of falls |
| Call staff nurse for resident examination | Refers to calling/informing staff nurses about a resident who has fallen, in order to assess residents |
| Do not move residents | Refers to avoiding the movement of residents who have fallen until assistance arrives, with nurses completing the examination, avoiding more injuries, and so on |
| Call an ambulance for transfer to hospital | Refers to calling emergency services/an ambulance to transfer to an acute hospital if needed |
| First aid | Refers to providing first aid and the treatment of injuries wound |
| Neurological assessment | Refers to conducting neurological observations involving the assessment of residents' cognition and consciousness |
| Vital signs assessment | Refers to assessing/evaluating vital signs, blood pressure, pulse, hypertension and hypotension |
| Pain assessment and management | Refers to identifying, assessing and treating pain |
| Fall circumstances assessment | Refers to knowing how the fall happened, along with the location and time of the fall |
| Safe transfer of residents by staff | Refers to using staff for transferring residents back to beds, chairs and rooms |
| Assessing safety for transfer and mobility | Refers to discussing, assessing and deciding how to move/transfer residents safely from a floor to a bed/chair by using a hoist/assistant, and so on |
| Ensure the surrounding environment is safe | Refers to keeping the area around the faller safe, clearing hazards and obstacles, and checking environmental safety before transferring and mobilising residents |
| Follow nurse instructions or policy | Refers to following the hospital/site/falls policy after a fall or to following nurses' instructions after assessing fallers to determine how to move them |
| Monitor residents | Refers to the monitoring of residents who recently experienced a fall, monitoring them within 24 hours or 48 hours, or via hourly checks, along with keeping close to residents to avoid falls and their consequences |
| Complete fall report | Refers to completing reports concerning fall incidents, risk assessments and documents such as post-fall logs, NIMS forms, FRSAE forms relating to fallers |
| Update care plan | Refers to updating the care plan and the fall risk assessment regarding fall incidents and faller assessments |
| Inform doctors or GPs | Refers to informing doctors, GPs or SouthDoc about fall incidents |
| Inform the authorised staff | Refers to informing/documenting/reporting to the senior nurse, manager, CNM, administrative nurse and DON regarding fall incidents |
| Refer to physio | Refers to referrals/reviews/assessments by physios |
| Inform all staff | Refers to telling all staff in the organisation about fall incidents and residents who have fallen |
| Inform family/relatives | Refers to informing the families/relatives of residents (KIN, NOK) about fall incidents |
| MDT fall analysis | Refers to discussing the causes of falls with staff, particularly at MDT meetings, to analyse the reasons for falls |
| Keep residents comfortable | Refers to making residents comfortable in terms of body position (head), lying on the floor and putting pillows under their heads |
| Reassure residents | Refers to reassuring residents, speaking/staying with them and keeping them calm |
| Protect the residents’ dignity | Refers to any action that mainly serves to protect the resident’s dignity |
| Staff peer learning | Refers to discussing or educating staff and sharing ideas and information with others |
| Staff collaboration | Refers to teamwork and having MDT meetings to support each other |
| Fall champions | Refers to having a co-ordinator/champion to support the staff in the workplace |
