# Supplementary material for: Staff’s insights into fall prevention solutions in long-term care facilities: a cross-sectional study
Source: BMC Geriatr. 2023 Nov 13;23:738. doi: 10.1186/s12877-023-04435-7 (PMC10644547; doi:10.1186/s12877-023-04435-7)
Supplement: Supplementary file 3 — Additional file 3: Supplementary file 3. Participant demographics. [file 12877_2023_4435_MOESM3_ESM.docx]

Supplementary file 3: Participant demographics

| Demographics | | | The survey; N=155 (100%) | |
| --- | --- | --- | --- | --- |
|  |  |  | N | (%) |
| Gender | M | | 31 | 20 |
|  | F | | 122 | 78.7 |
|  | Prefer not to say | | 2 | 1.3 |
| Age | 18-29 Y | | 28 | 18.1 |
|  | 30-39 y | | 43 | 27.7 |
|  | 40-49 Y | | 35 | 22.6 |
|  | 50-59 Y | | 36 | 23.2 |
|  | 60-65 Y | | 9 | 5.8 |
|  | Prefer not to say | | 3 | 1.9 |
|  | Missing | | 1 | 6 |
| Education level | FETAC level | | 40 | 25.8 |
|  | Bachelor | | 67 | 44.4 |
|  | Post-graduate Certificate | | 6 | 3.9 |
|  | Post-graduate Diploma | | 21 | 13.5 |
|  | Master | | 15 | 9.7 |
|  | Other | | 2 | 1.3 |
|  | Missing | | 4 | 2.6 |
| Job Role | Senior Nurse/CNM | | 32 | 20.9 |
|  | Nurse | | 51 | 32.9 |
|  | HCAs | | 55 | 35.5 |
|  | GP | | 8 | 5.2 |
|  | HSCP | | 2 | 1.3 |
|  | Other | | 5 | 3.2 |
|  | Missing | | 2 | 1.3 |
| Experience as a (paid) carer for older people | < a year | | 25 | 16.1 |
|  | 1-2 Y | | 20 | 12.9 |
|  | 3-5 y | | 24 | 15.5 |
|  | 6-10 y | | 18 | 11.6 |
|  | > 11 Y | | 66 | 42.6 |
|  | Missing | | 2 | 1.3 |
| Experience in their long-term care facility | 3-6 M | | 21 | 13.5 |
|  | 7-12 M | | 14 | 9 |
|  | 1-2 Y | | 34 | 21.9 |
|  | 3-5 y | | 22 | 14.2 |
|  | 6-10 y | | 21 | 13.5 |
|  | >11 Y | | 43 | 27.7 |
| Works shifts | One shift | Morning (7) | 72 | 46.5 |
|  |  | Full day (12 hours) (53) |  |  |
|  |  | Night (12 Hours) (8) |  |  |
|  |  | Twilight hours (4) |  |  |
|  | Two shifts | Morning/ Afternoon (16) | 50 | 32.3 |
|  |  | Morning/ Full day (1) |  |  |
|  |  | Full day/ Night (23) |  |  |
|  | Three shifts | Morning/ Afternoon/ Full day (7) | 14 | 9 |
|  |  | Morning/ Full day/ Night (7) |  |  |
|  | Four shifts | Morning/ Afternoon/ Full day/ Night (3) | 5 | 3.2 |
|  |  | Morning/ Afternoon/ Full day/ Twilight (2) |  |  |
|  | Five shifts | Morning/ Afternoon/ Full day/ Night/ Twilight | 5 | 3.2 |
|  | Missing | | 9 | 5.8 |

M: male; F: female; Y: year; FETAC: Further Education and Training Awards Council; CNM: Certified Nurse Midwife; HCAs: Healthcare assistants; GP: General practitioner; HSCP: Health social and care professional; M: month
