# Supplementary material for: Staff’s insights into fall prevention solutions in long-term care facilities: a cross-sectional study
Source: BMC Geriatr. 2023 Nov 13;23:738. doi: 10.1186/s12877-023-04435-7 (PMC10644547; doi:10.1186/s12877-023-04435-7)
Supplement: Supplementary file 4 — Additional file 4: Supplementary file 4. Fall prevention training – value, content and implementation of training into practice. [file 12877_2023_4435_MOESM4_ESM.docx]

Supplementary file 4: ***Fall prevention training – value, content and implementation of training into practice***

| ***Table 1: “What would help you use training you have received in practice to prevent falls when you are at work?”*** | | | | | | | |
| --- | --- | --- | --- | --- | --- | --- | --- |
| Categories | | | Codes | | | Quotes from healthcare workers | |
| Staff education/ training and self-efficacy | Fall prevention training is key for staff (n=54) | | Recognition of the value of education/ training (n=12) | | | “All training helps me in my work going forward  ” (Health worker 79)  “The training would ensure that I am better able to identify the risk of falls to ensure that residents are equipped with aids that would prevent falls.” (Health worker 94) | |
|  |  |  | Having **manual handling Training (n=11)** | | | “Proper moving and handling techniques and more awareness of patient safety” (Health worker 38)  “More emphasis on manual handling training” (Health worker 11)  “On how to handle in case of a fall scenario” (Health worker 93) | |
|  |  |  | On-site training (n=5) | | | “On-site practical training following education is more beneficial” (Health worker 153) | |
|  |  |  | Compulsory staff training (n=5) | | | “All staff to have the same level of education in falls prevention” (Health worker 41) | |
|  |  |  | **Ongoing training** (n=4) | | | “Retraining part of ongoing in-house training, all the training” (Health worker 127) | |
|  |  |  | Educational materials/ resources (i.e., videos, leaflets, lectures, and posters) (n=4) | | | “Posters with simple ideas/steps to prevent falls” (Health worker 113)  “Lecture in some videos” (Health worker 106)  “Copy of learning material to refer back to” (Health worker 145) | |
|  |  |  | New staff induction training) (n=4) | | | “Fall prevention should be mandatory training of staff before starting to work; we are dealing with life” (Health worker 106)  “To highlight the importance of training and education for new staff, especially” (Health worker 129) | |
|  |  |  | Translating training into practice (n=4) | | | “Being able to apply the training to a separate case” (Health worker 129) | |
|  |  |  | Adequate staff training (n=4) | | | “Provided adequate falls prevention training” (Health worker 87) | |
|  |  |  | Staff awareness (n=3) | | | “Awareness of high-risk fall residents” (Health worker 84)  “More understanding of safe practices and more knowledge about the cause and risk of falls” (Health worker 36) | |
|  |  |  | Understandable staff education (n=2) | | | “Course understandable” (Health worker 150) | |
|  |  |  | One-to-one training(n=1) | | | “More person-to-person training needed” (Health worker 100) | |
|  |  |  | Training delivered by an external trainer(n=1) | | | “We would greatly appreciate in-house training from others outside our facility” (Health worker 22) | |
|  | Staff self-efficacy and motivation (n=3) | | - Personal motivation(n=1) - Confidence Growth from training (n=2) | | | “In my opinion, each person must be protected in this area, no training is going to ensure that people will do the right thing, it has to come from the person within” (Health worker 114)  “More confidence in fall prevention and post-fall management” (Health worker 108) | |
| Having implementation strategies in operation to support staff in their practice; (n=26) | | | **Staff communication / Staff peer learning** (n = 13) | | | “The knowledge to educate other staff confidently about what I learnt and what possible improvements can help in preventing falls” (Health worker 65)  “Staff can bounce ideas off each other” (Health worker 145)  “Discussion with other team members on some points” (Health worker 155)  “Interacting with staff and asking questions and asking how I can improve” (Health worker 9) | |
|  |  |  | Audit/ feedback (n = 7) | | | “Manual handling implementors are designated daily to pick up on bad habits and encourage good techniques: one person every shift, and these are 24-hour care facilities” (Health worker 19)  “Physio on site continues with audits” (Health worker 19)  “Pointing out incorrect procedures and behaviour could help” (Health worker 114) | |
|  |  |  | Regular staff reminders (n = 3) | | | “Continuous reminding of risks” (Health worker 114) | |
|  |  |  | Fall champions (n = 2) | | | “Follow up in practice, e.g., falls champions who have received extra training and can support staff on the floor” (Health worker 16) | |
|  |  |  | Staff collaboration (n = 2) | | - Staff teamwork (n = 3) - MDT approach (n=1) | “For all staff to have fall knowledge through training so as to successfully work as a team” (Health worker 87)  “Teamwork and multidisciplinary approach” (Health worker 24) | |
|  |  |  | a problem-solving approach (n = 1) | | | “We will get new knowledge; we can identify the risks easily and help to prevent falls through a problem-solving strategy” (Health worker 130) | |
|  |  |  | Root cause analysis (n = 1) | | | “Root cause analysis of each fall, particular recurrent fallers” (Health worker 39) | |
| Involvement of residents and families in fall prevention (n=5) | | | - Resident Awareness (n = 2) - Family Education (n = 1) - Communication between Staff and Residents (n = 1) - Resident Participating in Training (n = 1) | | | “Make residents aware” (Health worker 44)  “Families' perception of falls needs to be changed; they seem to want to point blame instead of trying to resolve or reduce the risk” (Health worker 64)  “Communication between staff and residents is vital” (Health worker 87)  “Any training in fall prevention should include encouragement to get residents moving and discussion of the risk paradox there” (Health worker 152) | |
| Fall prevention activities (n=28) | | | | | | - Fall risk assessment (i.e., quick screen, multifactorial assessment) (n = 10) - Providing appropriate equipment (e.g., Sensors mats (n = 9) - Knowing residents’ history and care plan (n = 5) - Appropriate Footwear/ Clothing (n = 4) - Safe and Decluttered Environment (n = 4) - Residents’ supervision (n = 4) - Regular review of residents (timely) (n = 2) - Effective exercise programme (n = 1) - Medication review (n = 1) - Reporting fall incidence (n = 1) | |
| **Table2: “Are there any other comments you would like to add about falls prevention in residential care facilities?”** | | | | | | | |
| Categories | | | | Codes | | | Quotes |
| LTCF staffing issues (n=13) | | Adequate staff level | | Staffing issues (n=11) | | | “One cannot ignore the ongoing resource issues that influence falls, particularly resident: staff ratios” (Health worker 16) |
|  |  |  |  | Staffing ratio in HSE/non-HSE (n=2) | | | “A huge divide between what the HSE homes are provided with versus the private/ section 39 homes (….). staffing levels are regulated staff: residents’ ratio is not the same in non-HSE homes varies varied and diverse”. (Health worker 19) |
|  |  |  |  | Consistent staff positioning (n=1) | | | “Stop moving staff between wards every single day” (Health worker 140) |
|  |  | Specialist care | | More PT/OT(n=1) | | | “More physio input and OT input required”. (Health worker 19) |
|  |  |  |  | Maintenance Staff input (n=1) | | | “Maintenance staff can only ensure equipment/environment is as safe as possible”. (Health worker 116) |
|  |  |  | | Adequate time (n=1) | | | “Enough time” (Health worker 128) |
| Enhancing residents' autonomy (n=4) | | Barriers to residents’ autonomy | | - Increased fear of falling/reduced movement (n=2) - Reduce residents’ autonomy(n=1) - Over-monitoring/ frustrated residents (n=1) - Healthcare litigation (restricting residents’ Mobility) (n=1) | | | “We reduce people’s autonomy to make choices and end up curtailing their movement for fear of a fall, Life is a risk! Physical restraints may be eliminated but constant monitoring must be very frustrating for our residents” (Health worker 15)  “Residents must not be confined by fear of falling” (Health worker 24)  “We must take risks and let frail residents mobilize but litigation is now becoming an issue in Irish Healthcare”. (Health worker 73) |
|  |  | Promoting resident’s autonomy and independence | | - Improve resident’s mobility/ Decrease resident’s fear of falling (n=3) - Improve residents’ autonomy (n=2) | | | “Falls cannot be eliminated while promoting quality of life. Residents must not be confined by fear of falling and should be encouraged with mobility” (health worker 24)  “There is inadequate activation of residents (…) Any training in fall prevention should include encouragement to get residents moving”. (Health worker 152) |
| Cohorting high-risk residents (n=1) | | | | Residents’ group pods (n=1) | | | “In high-dependant units, I would like to see pods of residents with a career. Group quieter residents together to prevent them from being upset by more demanding residents” (Health worker 10) |
| Falls highlighted as the key challenge by staff (n=3) | | | | | | | “It is difficult, and the only way to prevent falls 100% is to provide 1;1 care to fall-risk residents, which is not possible!”. (Health worker 145)  “Very challenging problem” (Health worker 41) |
